# Characterization of *Neurospora crassa* GH16, GH17, and GH72 gene families of cell wall crosslinking enzymes

**DOI:** 10.1016/j.tcsw.2022.100073

**Published:** 2022-01-15

**Authors:** Pavan Patel, Stephen J. Free

**Affiliations:** Dept. of Biological Sciences, SUNY University at Buffalo, Buffalo, NY 14260, United States

**Keywords:** Chitin, Chitin transferase, β-1, 3-glucan, Glucan transferase, GH16, GH17, Fungal Cell Wall

## Abstract

GH16 chitin transferases, GH17 β-1,3-glucan transferases, and GH72 β-1,3-glucan/lichenin transferases are important fungal cell wall crosslinking enzymes. The *Neurospora crassa* genome encodes three genes from the GH17 gene family and five members in the GH16 subfamily 18 and 19 fungal chitin transferases. We created deletion mutants lacking all three GH17 genes and determined that they had wild type morphology and are more sensitive to cell wall perturbation reagents than the wild type. We also created deletion mutants lacking all five GH16 subfamily 18 and 19 genes and found that they had wild type morphology and are more sensitive to cell wall perturbation reagents than the wild type. We conclude that GH16 and GH17 enzymes play roles in cell wall biogenesis. In *N. crassa*, GH72 enzymes have been reported to be lichenin transferases, while in other fungi they have been shown to be the β-1,3-glucan transferases. Neurospora triple GH72 deletions give rise to a tight colonial morphology, sensitivity to cell wall perturbation reagents, and release of cell wall proteins into the medium. To ask if GH72 and GH17 enzymes might be redundant in *N. crassa*, we created sextuple mutants lacking the three GH72 genes and the three GH17 genes and found that they were indistinguishable from the GH72 triple mutant. We also found that a recombinant GH72 enzyme is able to form a lichenin-enzyme intermediate demonstrating that GH72 enzymes are lichenin transferases. The *N. crassa* GH72 enzymes are lichenin transferases and are not redundant with the GH17 β-1,3-glucan transferases.

## Highlights

• Mutants lacking GH16 chitin transferases are sensitive to cell wall perturbation reagents.
• Mutants lacking GH17 β-1,3-glucan transferases are sensitive to cell wall perturbation reagents.
• In N. crassa, GH17 β-1,3-glucan transferases and GH72 β-1,3-glucan/lichenin transferases are not redundant activities.
• Neurospora GH72 enzymes form lichenin-enzyme intermediates.
• Neurospora GH72 enzymes are lichenin transferases.

## 1 Introduction

Fungal cell walls are crosslinked matrices containing chitins, β-1,3-glucans, mixed β-1,3/1,4-glucans, β-1,6-glucans, α-glucans, mannans/galactomannans, and glycoproteins (Free, 2013, Klis et al., 2009, Lesage and Bussey, 2006). While β-1,3-glucan is found in virtually all fungal cell walls, the presence of the other polysaccharides varies from one fungus to another. The polysaccharide polymers constitute 80% to 85% of the cell wall mass, while glycoproteins constitute the remaining 15% to 20%. The fungal cell wall is an important organelle that protects the cell from various environmental stresses. The cell wall glycoproteins are required for vital functions, like adhesion, signal transduction, biofilm formation, and cell wall biosynthesis. Fungal pathogens have become a major problem for patients having immunological deficiencies, and drug resistance arising in pathogenic fungi has prompted the search for new antifungal strategies. Since the fungal cell wall is vital for the survival of the fungi and is a unique structure present only in fungal cells, it represents an ideal target for the development of new anti-fungal agents.

Mutants of chitin synthases, β-1,3-glucan synthases, and α-1,3-glucan synthases demonstrate the importance of these polysaccharide polymers for cell wall biogenesis (de Groot et al., 2009, Free, 2013, Fu et al., 2014, Gow et al., 2017). These transmembrane polysaccharide synthases are located on the plasma membrane, where they add sugar residues to the non-reducing ends of the polysaccharides and extrude the polysaccharides into the cell wall space during synthesis. The extruded polysaccharides are subsequently crosslinked together within the cell wall space by enzymes having glucan and chitin transferase activities (Cabib et al., 2007, Cabib et al., 2008, Goldman et al., 1995, Mouyna et al., 2000). Members of the GH16, GH17, and GH72 families of chitin, β-1,3-glucan, and β-13-glucan/lichenin transferases have been frequently identified in proteomic and RNAseq expression analyses of fungal cell walls. Each of these crosslinking enzyme families have multiple members encoded in all characterized fungal genomes. Different combinations of these crosslinking enzymes from each of the multigene families are expressed in the various cell types formed during fungal life cycles. This creates a situation of redundancy in GH16, GH17, and GH72 enzymatic activities. This functional redundancy can help the fungi grow in varying environmental conditions (Fonzi, 1999). These crosslinking enzymes carry out two closely related catalytic activities as they function to crosslink cell wall polysaccharides together. First, they function as glycosylhydrolases to cleave chitin or β-1,3-glucan polymers near their reducing ends. This reaction generates an enzyme-polysaccharide intermediate with a covalent bond between the reducing end of the cleaved polysaccharide and a glutamate or aspartate residue in the active site of the enzyme. In the second reaction, the enzymes act as polysaccharide transferases. In this reaction, they transfer the attached polysaccharide to the non-reducing end or to an internal site on a second polysaccharide. This generates a new glycosidic bond and creates a crosslinked polysaccharide cell wall matrix.

Multiple GH16, GH17, and GH72 enzymes have been identified in the *Neurospora crassa* cell wall (Bowman et al., 2005, Maddi et al., 2009, Maddi and Free, 2010). The *N crassa* genome encodes 15 genes from GH16 family, 3 genes from GH17 family, and 5 genes from GH72 family. The CAZY website contains nearly 23,000 genes in the GH16 family of glycosylhydrolases which have been assigned to 23 subfamilies (Viborg et al., 2019). GH16 sister subfamilies 18 and 19 encode fungal-specific chitin transferases. The GH16 subfamilies 18 and 19 include *Saccharomyces cerevisiae* Crh1p, Crh2p, and Crr1p, *Candida albicans* Crh11, Crh12, and Utr2, and *Aspergillus fumigatus* Crh1 and Crh5. *S. cerevisiae* Crh1p and Crh2p have the ability to use chitin oligosaccharides as donors and to transfer them to the non-reducing ends of β-1,3-glucans, β-1,6-glucans, and to other chitin oligosaccharides (Arroyo et al., 2016, Blanco et al., 2015, Cabib et al., 2007, Cabib et al., 2008, Gomez-Esquer et al., 2004, Mazan et al., 2013, Pardini et al., 2006). The *A. fumigatus* Crh1 and Crh5 enzymes have been characterized and shown to transfer chitin oligosaccharides to the non-reducing ends of β-1,3-glucan and chitin oligosaccharides (Fang et al., 2019). The crystal structure of the *A. fumigatus* Crh5 has been characterized with an associated chitin oligosaccharide and several amino acids that play key roles in substrate binding and catalysis have been identified (Fang et al., 2019).

GH17 family enzymes are capable of connecting two β-1,3-glucans together through a β-1,6-linkage (Aimanianda et al., 2017, Gastebois et al., 2010, Goldman et al., 1995, Kutty et al., 2019, Mouyna et al., 1998). GH17 enzymes have been shown to attach a donor β-1,3-glucan to the non-reducing end of an acceptor β-1,3-glucan through a β-1,6-linkage to generate a “kinked” linear glucan. They have also been shown to attach a donor β-1,3-glucan to an internal site of the acceptor β-1,3-glucan through a β-1,6-linkage to generate a branched glucan (Aimanianda et al., 2017, Gastebois et al., 2010).

The GH72 family enzymes have been extensively studied in *Saccharomyces cerevisiae*, *Candida albicans*, and *Aspergillus fumigatus*, where they have been shown to act as β-1,3-glucan hydrolases and transferases. In these fungi, the GH72 enzymes have been shown to be able to add β-1,3-glucan in a β-1,3- linkage to the non-reducing end of a second β-1,3-glucan or to add β-1,3-glucan to an internal site on a second β-1,3-glucan through a β-1,6-linkage (Aimanianda et al., 2017, Gastebois et al., 2010, Goldman et al., 1995, Mouyna et al., 1998). The combined activities of a GH17 and a GH72 enzyme are thought to be responsible for generating the β-1,6-branched β1-3-glucan matrix in *S. cerevisiae* (Aimanianda et al., 2017). The GH72 enzymes have also been studied in *N. crassa* (Ao and Free, 2017, Patel and Free, 2019). Interestingly, in *N. crassa* the GH72 enzymes function as lichenin (mixed β-1,3-/β-1,4-glucan) transferases, and are used to form crosslinks between lichenin and a galactomannan structure found on cell wall glycoproteins (Kar, Patel, Ao, & Free, 2019). In carrying out these reactions, the *N. crassa* GH72 enzymes crosslink cell wall glycoproteins into the cell wall. The combined crosslinking activities of the GH16, GH17, and GH72 polysaccharide transferases have the capacity to provide for the formation of the crosslinked polysaccharide matrix characteristic of the fungal cell wall.

Genetic analyses of the GH16 and GH17 gene families have been carried out in *C. albicans*, *S. cerevisiae*, and *A. fumigatus*. Genetic analyses in which multiple GH16 genes have been deleted have shown relatively mild phenotypes, suggesting minor alterations in cell wall structure and strength (Cabib et al., 2007, Gomez-Esquer et al., 2004, Pardini et al., 2006, van Leeuwe et al., 2020). Similarly, genetic analyses in which multiple GH17 genes have been deleted have shown relatively mild phenotypes (Sarthy et al., 1997, Sestak et al., 2004). For example, the double mutant of *A. fumigatus* GH17 enzymes AfBGT1 and AfBGT2 does not exhibit any cell wall defects (Gastebois et al., 2010).

To characterize the *N. crassa* GH17 genes, we generated all combinations of single, double, and triple deletion mutants for the three GH17 genes. While all of the single and double mutants had a wild type phenotype, we found that the triple deletion mutants had a mild growth phenotype and were sensitive to cell wall perturbation reagents. This indicates that the three GH17 enzymes have redundant functions. Our results are similar to the results for deletions of GH17 genes in other fungi. We also characterized the GH16 subfamily 18 and 19 chitin transferases by creating different combinations of deletion mutants. We demonstrated that mutants lacking all 5 of the GH16 subfamily 18 and 19 enzymes have a mild growth defect and are sensitive to cell wall perturbation reagents. We conclude that the five GH16 chitin transferases have redundant activities and function in cell wall biosynthesis but are not essential for cell viability. These findings are similar to the mild phenotypes seen in *S. cerevisiae*, *C. albicans*, *A niger*, and *A. fumigatus* GH16 deletion mutants (Cabib et al., 2007, de Groot et al., 2009, Fang et al., 2019, Gomez-Esquer et al., 2004, Pardini et al., 2006, van Leeuwe et al., 2020).

In *S. cerevisiae*, GH17 and GH72 β-1,3-glucan transferases have been shown to have redundant activities in crosslinking the cell wall β-1,3-glucans and mutants with deletions in both families of enzymes are drastically sick (Aimanianda et al., 2017). The *N. crassa* GH72 enzymes have been reported to function as lichenin transferases (Kar et al., 2019). In *N. crassa*, triple deletion GH72 mutants have been shown to grow in a tight colonial morphology and are very sensitive to cell wall perturbation reagents. To determine if the *N. crassa* GH17 and GH72 enzymes might have redundant β-1,3-glucan transferase activities, we created a sextuple mutant lacking the three GH72 genes and the three GH17 genes. We found that this mutant was indistinguishable for the GH72 triple mutant, suggesting that the *N. crassa* GH17 and GH72 enzymes are not redundant and corroborating the report that *N. crassa* GH72 is a lichenin transferase. To further characterize the *N. crassa* GH72 enzymes, a recombinant GH72 enzyme, GEL-2, was produced in *E. coli* and shown to form an enzyme-lichenin intermediate, demonstrating that the enzyme is a lichenin transferase.

## 2 Materials and Methods

### 2.1 Materials, strains and growth conditions

Single gene deletion mutant strains and wild type strains were obtained from the Fungal Genetics Stock Center (Manhattan, KS, USA). The single gene deletion mutants for members of the GH16 subfamilies 18 and 19 were *Δgh16-5/Δncu05686 (subfamily 19)*, *Δgh16-7/Δncu05974 (subfamily18)*, *Δgh16-10/Δncu08072 (subfamily 18)*, *Δgh16-11/Δncu09117 (subfamily 18)*, and *Δgh16-14/Δncu09672 (subfamily 18)*. The single gene deletion mutants for the GH17 family were *Δgh17-1/Δncu06381*, *Δgh17-3/Δncu09175*, and *Δgh17-4/Δncu09326*. Strains carrying up to five deletions within the GH16 gene subfamily 18 and 19 and strains with up to three deletions within the GH17 gene family were generated by classical genetics mating procedures (Davis & DeSerres, 1970). Different combinations of the GH16 deletion mutants *Δgh16-5*, *Δgh16-7*, *Δgh16-10*, *Δgh16-11*, and *Δgh16-14* were mated to generate double, triple, quadruple, and quintuple mutants. Pairs of single mutants were used to generate double mutants. Pairs of double mutants were used to create triple mutants. Pairs of triple mutants were used to create quadruple mutants. Pairs of quadruple mutants were used to create quintuple mutants. Similarly, combinations of the different GH17 deletion mutants were mated to create double and triple GH17 gene deletion mutants. To identify mutant isolates carrying multiple deletion mutations, genomic DNA from individual ascospore progeny from these matings were isolated and PCR reactions were used to assess the presence of the wild type and deletion alleles. Vogel’s sorbose medium, which causes *N. crass*a to grow in a tight colonial manner, was used to isolate individual ascospore progeny from these matings (Davis & DeSerres, 1970). A phylogeny tree for the GH16 enzymes was constructed using SeaView version 5 and FigTree tools (Gouy, Tannier, Comte, & Parsons, 2021).

To obtain sextuple deletion mutants lacking three GH17 genes and three GH72 genes, we crossed a mutant with three GH17 gene deletions with a strain with deletions of three of the GH72 genes. GH72 mutants have been previously characterized as having a tight colonial growth phenotype, sensitivity to cell wall perturbation reagents and as releasing their cell wall proteins into the growth medium (Ao & Free, 2017). Individual ascospore progeny having a tight colonial growth phenotype were isolated and PCR reactions were used to identify mutants having all six deletion mutations. The PCR reactions described below were used in determining whether progeny isolates contained the deletion allele or the wild type allele of each gene segregating within a cross.

### 2.2 PCR amplification and primers

To verify the presence of GH16, GH17, and GH72 gene deletions, PCR amplifications of wild type and mutant alleles of the genes were done using Taq polymerase. The GH16, GH17, and GH72 deletion alleles were originally generated by homologous recombination between the genomic DNA and a DNA construct with the hygromycin-resistance cassette between sequences upstream and downstream of the coding region. Two sets of primers were used to determine whether the wild type allele or deletion allele was present for each of the GH16 or GH17 genes in the progeny from the matings used to create the multiple gene deletion mutants. The first set of primers was used to amplify the wild type copy of the gene. This set of primers included a primer from the genomic region upstream of the hygromycin insertion site and a second primer from within the gene coding region. The second set of primers was used for the amplification of the deletion allele. This set of primers included the primer from the genomic region upstream of the hygromycin insertion site and a second primer from within the hygromycin coding region. Primers were provided by Integrated DNA Technologies (Coralville, IA). Table 1 gives the sequences of the hygromycin primer and the gene specific primers. Genomic DNA from each of the characterized haploid progeny isolates was PCR amplified with both sets of primers to determine whether the progeny had the deletion or wild type copy of the GH16, GH17, or GH72 family gene.

**Table 1:** Primers used to verify the presence of GH16, GH17, and GH72 gene deletion alleles.

Gene name	Sequence name	Sequence
GH17-1 ncu06381	6381F	CATCGTGTTTGCTATCCCGTC
GH17-1 ncu06381	6381 R	GCAGCCAAAGCTACGAGTTGC
GH17-3 ncu09175	9175F	CTCGCTCCATCTTCATCTTGC
GH17-3 ncu09175	9175 R	CCTGGATGTTGGTGTAGAGAC
GH17-4 ncu09326	9326F	AGGATGGATCGAGCTTGTGGC
GH17-4 ncu09326	9326 R	AAGAGTGAAGCGGTGGTGTGG
GH16-5 ncu05686	5686F	TTTCTGAAGCCAACCACCTGC
GH16-5 ncu05686	5686 R	ACTTACGAGAACAGCAAGGCG
GH16-7 ncu05974	5974F	ATGGACATGGACATGGAGGAG
GH16-7 ncu05974	5974 R	TGTCGTACTGGAGAGTAGTGC
GH16-10 ncu08072	8072F	TACCGATCCCACGATCACCCA
GH16-10 ncu08072	8072 R	AGGCCCAGGCTTTGGGAGTAG
GH16-11 ncu09117	9117F	GGCAAGCGAGTGTCATGCAAG
GH16-11 ncu09117	9117 R	GGACGGTTAGCGTTAGGCATG
GH16-14 ncu09672	9672F	GTCTTACGGACACGATACAGC
GH16-14 ncu09672	9672 R	TGATTGTGCAGCAGCCAACGC
GEL-1ncu08909	8909F	TAGAGGTCTTTCGCTACTGCC
GEL-1Ncu08909	8909 R	GTGGAATCGAGTGTGGTTAGC
GEL-2ncu07253	7253F	TCTTGGATGGTGGCTATGCAC
GEL-2ncu07253	7253 R	GTGATGGAAGCACGCTTGGTG
GEL-5 ncu06781	6781F	ATTCCTGACACTCGTTCGTCC
GEL-5 ncu06781	6781 R	CAACGTTCACATGCGTGCCAC
Hygromycin resistance	Hygro#2(Reverse)	GGCTGTGTAGAAGTACTCGCC

### 2.3 Phenotype screening, growth rate assay, and cell wall stress tests

To determine the radial growth rate of the mutant strains, conidia were inoculated at the edge of Vogel’s 2% sucrose 2% agar plates and the extension of leading edge of the hyphae was measured as a function of time. To assess if the mutants had weakened cell walls, a series of cell wall stress tests were performed in triplicate. The cell wall stress reagents used were the β-glucan synthase inhibitor caspofungin (up to 10 µg/ml), high salt (up to 9% NaCl), Calcofluor white (up to 10 mg/ml), and SDS (up to 0.01%) (Ao and Free, 2017, Maddi and Free, 2010). Caspofungin was a kind gift from Merck Research Laboratories (Rahway, NJ). Plates with varying concentrations of each of these reagents in Vogel’s sucrose agar were inoculated with a spot of wild type or deletion mutant conidia. The extension of the hyphae across the agar medium was measured as a function of time to give a growth rate. An average growth rate with a standard deviation was determined for the wild type and multiple GH16 sextuple and GH17 triple mutant isolates. The growth rates for wild type and mutant isolates on plates with perturbation reagents were compared with the growth rate of the same strains on control Vogel’s sucrose plates to give a percentage of the control growth rate in the presence of the perturbation reagent.

### 2.4 Western blot analysis of the recombinant GH72 enzyme-lichenin intermediate

To determine if the GH72 enzyme GEL-2 could function as a lichenin transferase, we generated a chimeric recombinant MBP::GEL-2 gene (a chimeric protein with a maltose binding protein N-terminus fused with the GEL-2 coding sequence that had been optimized for expression in *E. coli*). The codon-optimized version of *gel-2* (NCU07253) was designed and produced by Genewiz (South Plainfield, NJ). The recombinant *gel-2* gene was inserted into the pMal-p5X vector (New England Biolabs, Ipswitch, MA) for production of the chimeric MBP::GEL-2 protein. The recombinant protein was expressed in DH5α cells and purified on an amylose (MBP binding) column from New England Biolabs using the manufacturer’s protocol. To assay for the formation of a GEL-2-lichenin intermediate, 4 µg of purified GEL-2 and 50 µg of lichenin (InvivoGen, San Diego, CA) were incubated together in 20 µl of 50 mM sodium acetate pH 5.0 buffer at 30 °C for 5 min. The assay samples were then subjected to SDS PAGE. After transferring the protein from the gel to a nitrocellulose membrane, a Western blot assay was performed using a monoclonal anti-lichenin antibody (Australia Biosupplies, Victoria, Australia) and an HRP-conjugated goat anti-mouse antibody (Jackson ImmunoResearch Laboratories, Inc. West Grove, PA). The ECL kit (BioRad, Hercules, CA) was used to visualize the presence of lichenin associated with the recombinant GEL-2 protein.

## 3 Results

### 3.1 Isolation and characterization of GH17 deletion mutants

**Fig. 1:**
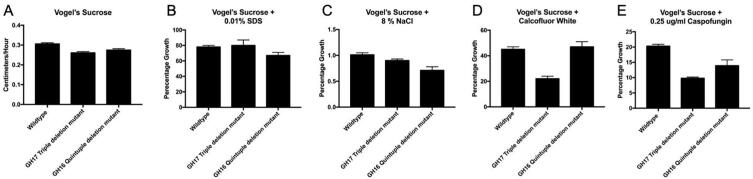
Loss of GH16 and GH17 crosslinking enzymes results in a cell wall defect. The radial growth rates of the wild type, a GH16 quintuple mutant, and a GH17 triple mutant were assessed on Vogel’s sucrose agar medium (panel A) and on Vogel’s sucrose agar medium supplemented with 0.01% SDS (panel B), 8 % NaCl (panel C), 10 mgr/ml Calcofluor White (panel D), and 0.25 µgr/ml caspofungin (panel E). The growth rates of the three isolates on Vogel’s sucrose medium is given in cm/hr (Panel A). The growth rates for the isolates in the presence of the cell wall perturbation reagents (Panels B, C, D, and E) is given as the % of that isolate’s growth rate on the control Vogel’s sucrose medium. The standard deviation for each of the measurements is shown as an error bar. The GH16 quintuple mutant and the GH17 triple mutant show sensitivity to presence of the cell wall perturbation reagents.

The GH17 family enzymes play an important role in crosslinking β-1,3-glucans together through β-1,3- and β-1,6-glycosidic bonds (Aimanianda et al., 2017, Gastebois et al., 2010, Goldman et al., 1995, Mouyna et al., 1998). The *N. crassa* genome encodes three GH17 family β-1,3-glucan transferases, GH17-1, GH17-3, and GH17-4. To characterize the role that these enzymes play during cell wall formation, we used standard *N. crassa* mating experiments to isolate every combination of single, double and triple GH17 mutants. The presence of the deletion alleles in these mutants was assessed by PCR amplification experiments as described in the Materials and Methods (see Figure S1). In characterizing these mutants, we found that all of these mutants had a wild type morphology when grown on Vogel’s sucrose medium. The mutants were also completely normal in conidiation and in sexual development. Our findings are in agreement with a previous study in which *N. crassa* GH17 single and double mutants were characterized (Martinez-Nunez & Riquelme, 2015). However, while characterizing the triple mutant, we found that it grew slightly slower than the wild type. We also found that the triple mutant was more sensitive than the wild type to the presence of cell wall perturbation reagents. In particular, we found that the triple mutant had a larger reduction in its radial growth rate than the wild type in the presence of 8% NaCl, 10 mg/ml Calcofluor White, and 0.25 µg/ml caspofungin (Fig. 1). Previously conducted RNAseq expression experiments indicate that *gh17-3* and *gh17-4* are expressed during vegetative growth, while *gh17-1* expression is associated with the sexual reproductive part of the life cycle (Liu et al., 2017). Our results indicate that the GH17 enzymes are redundant in their activities and are playing a role in cell wall biogenesis.

### 3.2 Isolation and characterization of GH16 deletion mutants

**Fig. 2:**
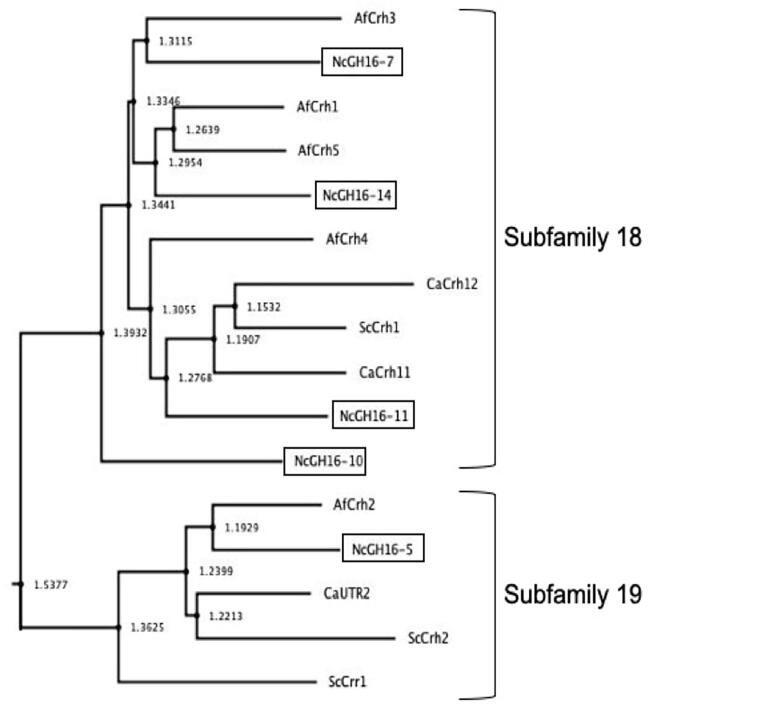
A phylogenetic tree for the GH16 family genes included in this study along with their orthologs from *S. cerevisiae*, *C. albicans*, and *A. fumigatus* was constructed using SeaView version 5 (Gouy et al., 2021). The five *N. crassa* enzymes are given with an Nc prefix and shown in boxes, the *S. cerevisiae* enzymes with a Sc prefix, the *C. albicans* enzymes with a Ca prefix, and the *A. fumigatus* enzymes with an Af prefix.

The CAZY GH16 family has been subdivided into 23 subfamilies (Viborg et al., 2019). Subfamilies 18 and 19 contain fungal-specific chitin transferases (Crh enzymes) which have been shown to be involved in crosslinking chitin to β-1,3-glucans and to other chitin oligosaccharides (Blanco et al., 2015, Fang et al., 2019). The *N. crassa* genome contains five GH16 subfamilies 18 and 19 genes. A phylogenic tree with these enzymes, along with their orthologs from *S. cerevisiae*, *C. albicans*, and *A. fumigatus* is shown in Fig. 2. Pair-wise comparisons between the five *N. crassa* enzymes show that they have between 33% and 46% sequence identity and between 50% and 63% sequence similarity. To characterize the GH16 family enzymes, we used standard *N. crassa* mating experiments to generate strains with all combinations of double, triple, quadruple, and quintuple deletion mutants for the GH16 gene subfamily 18 and 19 members *gh16-5*, *gh16-7*, *gh16-10*, *gh16-11*, and *gh16-14*. PCR amplification experiments were used to assess the presence of the wild type and deletion alleles and identify mutants with the various combinations of mutant alleles. Figure S2 shows an example of a PCR amplification experiment to identify a mutant having deletion alleles of the five GH16 genes. In characterizing these mutants, we found that all of our combinations of GH16 deletion mutants, including the quintuple deletion mutant, were morphologically normal. These mutants were also normal in conidiation and sexual development. The quintuple mutants had a slower radial growth rate than the wild type when grown on Vogel’s sucrose agar plates (Fig. 1). The quintuple mutants were tested for growth in the presence of cell wall perturbation reagents and were found to be more sensitive than the wild type to 0.01% SDS, 8% NaCl, and 0.25 µg/ml caspofungin (Fig. 1). We conclude that the GH16 enzymes are needed to synthesize a normal cell wall and that the enzymes represent a redundant family of cell wall crosslinking activities.

### 3.3 Characterization of sextuple mutants lacking GH17 and GH72 enzymes

**Fig. 3:**
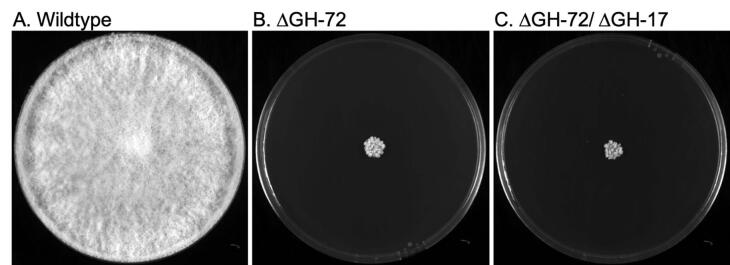
Morphology of GH72 triple mutants and the GH72/GH17 sextuple mutants. Conidia from wild type and mutant strains were inoculated in the middle of Vogel’s 2% sucrose agar plates and allowed to grow for 48 h before being photographed. Panel A shows the image of a wild type colony.Panel B shows the image of a GH72 triple mutant colony.Panel C shows the image of a GH72/GH17 sextuple mutant colony.

In *S. cerevisiae*, *A. fumigatus*, and *C. albicans*, the GH17 and GH72 enzymes have been shown to be β-1,3-glucan transferases, while the *N. crassa* GH72 enzymes have been reported as having lichenin (mixed β-1,3-/β-1,4-glucan) transferase activity (Kar et al., 2019). In *S. cerevisiae*, the simultaneous deletion of GH17 and GH72 enzymes has been shown to generate a “dramatically sick mutant phenotype”, indicating that GH17 and GH72 enzymes represent a redundancy in cell wall crosslinking activity (Aimanianda et al., 2017). We have previously characterized the GH72 triple mutant lacking GEL-1 (NCU08909), GEL-2 (NCU-07253), GEL-5 (NCU06781) and demonstrated that the mutant has a tight colonial growth morphology, releases cell wall proteins into the medium, and is sensitive to cell wall perturbation reagents (Ao & Free, 2017). We also demonstrated that the GH72 enzymes were able to mediate the transfer of lichenin onto a GH76 enzyme-processed N-linked galactomannan (Kar et al., 2019). However, we considered the possibility that the *N. crassa* GH72 enzymes might be able to utilize both lichenin (a mixed β-1,3/β-1,4-glucan) and β-1,3-glucan, two closely related polysaccharides, and function to crosslink both into the cell wall. If this were the case, *N. crassa* GH72 and GH17 enzymes would have redundant activities. To determine if the *N. crassa* GH17 and GH72 enzymes might be functionally redundant, we prepared and characterized a sextuple deletion mutant lacking all three GH17 genes and three GH72 genes as described in Materials and Methods. We found that the sextuple mutant was indistinguishable for the GH72 triple mutant in growth characteristics (Fig. 3) and in sensitivity to cell wall perturbation reagents. Specifically, we found no differences in their growth in 0.01% SDS, and caspofungin. Both mutants were unable to grow in the presence of 10 mg/ml Calcofluor White, and the addition of 0.8% NaCl to the medium gave a similar partial reversion of their colonial growth phenotype (Ao & Free, 2017). We found no evidence to suggest functional redundancy between the *N. crassa* GH17 and GH72 enzymes. This suggests that, unlike the situation in *S. cerevisiae*, the *N. crassa* GH72 enzymes do not have redundancy with the GH17 β-1,3-glucan transferases and that GH72 is likely to function solely as a lichenin transferase.

### 3.4 N. Crassa GEL-2 is able to form an enzyme-substrate intermediate with lichenin

**Fig. 4:**
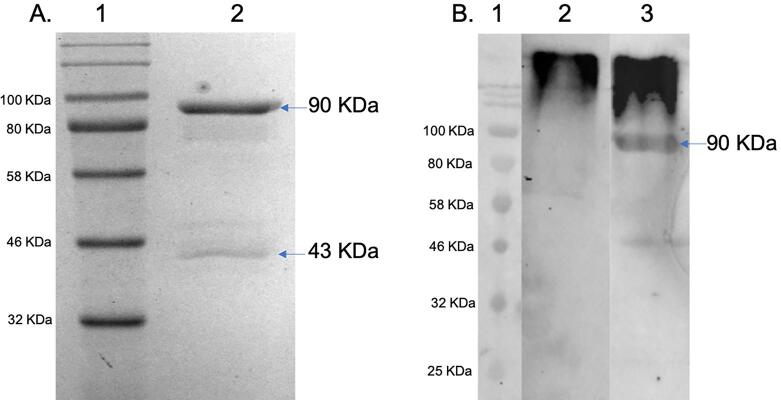
Purification of MBP::GEL-2 and Western blot analysis of a GEL-2-lichenin intermediate. Panel A shows a Coomassie-stained gel of MBP::GEL-2 purified on an amylose column. Lane 1 contains molecular weight markers and lane 2 shows the purified MBP::GEL-1. The major band of protein at 90 KDa represents the chimeric MBP::GEL-2 while the protein band at 43 KDa represents MBP without GEL-2. In panel B the purified recombinant MBP::GEL-2 was incubated with lichenin and then subjected to SDS PAGE and Western blot analysis with antibodies directed against lichenin. Lane 1 contains molecular weight markers. Lane 2 is a control lane containing the lichenin substrate without the chimeric MBP::GEL-2. Lane 3 is the experimental lane with lichenin and MBP::GEL-2. The presence of a lichenin-GEL-2 intermediate at 90 KDa is seen in lane 3. The lichenin substrate is located at the top of the gel and Western blot.

To further characterize the *N. crassa* GH72 enzymes, we prepared a recombinant chimeric maltose-binding protein/GEL-2 construct in which the GEL-2 coding sequence had been optimized for expression in *E. coli*. The construct was expressed and the 90 KDa MBP::GEL-2 chimeric protein was purified on an amylose column (Fig. 4 panel A). The purified MBP::GEL-2 and a control MBP sample were incubated together with lichenin and the ability of GEL-2 to form an enzyme-lichenin intermediate was assessed in a Western blot assay using a monoclonal anti-lichenin antibody. As seen in Fig. 4 panel B, the recombinant GEL2- was able to form an enzyme-lichenin intermediate with a molecular weight of 90 KDa, which can be seen at a position below the free lichenin, which remains near the top of the SDS gel during electrophoresis. We previously published the results of *in vitro* lichenin transferase experiments showing that the transfer of lichenin to cell wall glycoproteins was dependent upon the presence of the GH72 enzymes (Kar, Patel, Ao, & Free, 2019). Based on the results of our *in vitro* lichenin transferase experiments and the formation of an enzyme-lichenin intermediate, we conclude that the *N. crassa* GEL-2 is a lichenin transferase.

## 4 Discussion

Virtually all fungal genomes contain multiple genes from GH16 subfamilies 18 and 19 chitin transferases (Viborg et al., 2019). Fungal genomes also contain multiple GH17 β-1,3-glucan transferase genes and GH72 β-1,3-glucan/lichenin transferase genes encoding cell wall crosslinking activities. The available literature from *S. cerevisiae* and *A. fumigatus* shows that the GH16 chitin transferases from these fungi can use chitin or β-1,3-glucan as an acceptor polysaccharide (Blanco et al., 2015, Fang et al., 2019), and that the GH17 enzymes can use a second β-1,3-glucan as an acceptor (Aimanianda et al., 2017, Cabib et al., 2007, Goldman et al., 1995, Hartl et al., 2011, Mouyna et al., 1998). Proteomic and gene expression analyses have shown that multiple GH16, GH17, and GH72 enzymes are being expressed in the various types of cells formed during fungal life cycles. Paradoxically, genetic analyses have shown that deletion mutants lacking GH16 and GH17 enzyme families have rather mild phenotypes (Cabib et al., 2007, Cabib et al., 2008, Gomez-Esquer et al., 2004, Pardini et al., 2006, Sarthy et al., 1997, Sestak et al., 2004, van Leeuwe et al., 2020). In this study, we carried out a genetic analysis of these enzymes in the haploid fungus *N. crassa*. Our results corroborate the studies carried out in other fungi in demonstrating that *N. crassa* mutants in which the GH16 subfamilies 18 and 19 chitin transferase genes have been deleted have a mild growth phenotype and are sensitive to cell wall perturbation reagents (Fig. 1). We also found that mutants lacking the GH17 β-1,3-transferase enzymes have a mild growth phenotype (Fig. 1). The GH16 deletion mutants and the GH17 deletion mutants show clear differences in their sensitivity to the tested cell wall perturbation reagents (Fig. 1). This indicates that there are structural differences between the cell wall formed in the absence of GH16 chitin transferases and the cell wall formed in the absence of GH17 β-1,3-glucan transferases.

We also asked whether the GH17 β-1,3-glucan transferases and the GH72 β-1,3-/lichenin transferases might be redundant cell wall crosslinking enzymes. In *S. cerevisiae*, the simultaneous loss of both types of cell wall crosslinking enzymes generates a severely sick phenotype, demonstrating that the two crosslinking enzymes have functional redundant β-1,3-glucan transferase activity (Aimanianda et al., 2017). In *N. crassa*, GH72 deletion mutants have a tight colonial phenotype, are quite sensitive to cell wall perturbation reagents, and release their cell wall proteins into the medium (Ao & Free, 2017). We found that *N. crassa* mutants lacking GH17 and GH72 enzymes were indistinguishable from mutants lacking only the GH72 enzymes (Fig. 3) suggesting that the two types of crosslinking enzymes are not redundant in *N. crassa* and that the two types of enzymes have two distinct functions. We have previously shown that in *N. crassa* the GH72 enzymes can function as lichenin transferases to transfer lichenin onto processed galactomannan structures on the N-linked oligosaccharides present on cell wall glycoproteins (Kar et al., 2019). In this report we demonstrate that a recombinant GEL-2 (NCU07253) expressed in *E. coli* has the ability to form an enzyme-substrate intermediate with lichenin (Fig. 4) but not with β-1,3-glucan. This clearly demonstrates that *N. crassa* GH72 enzymes function as lichenin transferases. Based on the results presented in this report and in our previous publications, we hypothesize that the *N. crassa* GH72 enzymes function as lichenin transferases to crosslink the N-linked galactomannans found on cell wall glycoproteins into the cell wall (Ao and Free, 2017, Kar et al., 2019). The possibility remains that they may also be able to crosslink lichenin to other donors, such as chitin, β-1,3-glucans and/or lichenin polymers. Our results suggest that unlike the situation in *S. cerevisiae* where GH17 and GH72 enzymes have functional redundancy (Aimanianda et al., 2017, Gastebois et al., 2010, Goldman et al., 1995, Mouyna et al., 1998), the *N. crassa* GH72 lichenin transferases are not redundant with the GH17 β-1,3-glucan transferases.

## 5 Conclusions

We have carried out a genetic analysis of the roles that GH16 chitin transferases, GH17 β-1,3-glucan transferases, and the GH72 β-1,3-lichenin transferases play in the formation of the *N. crassa* cell wall. We have demonstrated that all three types of enzymes play roles in cell wall biogenesis. Loss of the GH16 subfamilies 18 and 19 fungal-specific chitin transferases generates a mild growth phenotype and sensitivity to cell wall perturbation reagents. Similarly, the loss of GH17 enzymes generates a mild growth phenotype and sensitivity to cell wall perturbation reagents. Loss of GH72 enzymes gives a much more dramatic tight colonial growth phenotype with sensitivity to cell wall perturbation reagents and the release of cell wall glycoproteins into the medium. Our results demonstrate that an *N. crassa* GH72 enzyme, GEL-2, can generate enzyme-lichenin intermediates, indicating that it functions as a lichenin transferase. Furthermore, our results provide no evidence for GH17 β-1,3-glucan transferases and GH72 lichenin transferases having a redundancy in their functional activities. Our results indicate that in *N. crassa* the three types of polysaccharide transferases carry out three distinct cell wall crosslinking functions.

## Declaration of Competing Interest

The authors declare that they have no known competing financial interests or personal relationships that could have appeared to influence the work reported in this paper.
